# Enhanced gastrointestinal survivability of recombinant *Lactococcus lactis* using a double coated mucoadhesive film approach

**DOI:** 10.1371/journal.pone.0219912

**Published:** 2019-07-23

**Authors:** Ee Wern Tan, Kean Yuen Tan, Li Voon Phang, Palanirajan Vijayaraj Kumar, Lionel L. A. In

**Affiliations:** 1 Department of Biotechnology, Faculty of Applied Sciences, UCSI University, Kuala Lumpur, Malaysia; 2 Department of Pharmaceutical Sciences, Faculty of Pharmaceutical Sciences, UCSI University, Kuala Lumpur, Malaysia; 3 Functional Food Research Group, Faculty of Applied Sciences, UCSI University, Kuala Lumpur, Malaysia; St. John’s University, UNITED STATES

## Abstract

Vaccine administration via the oral route is preferable to parenteral routes due to ease of administration. To date, most available oral vaccines comprises of live attenuated pathogens as oppose to peptide-based vaccines due to its low bioavailability within the gastrointestinal (GI) tract. Over the years, probiotic-based peptide delivery vehicles comprising of lactic acid bacteria such as *Lactococcus lactis* has emerged as an interesting alternative due to its generally recognized as safe (GRAS) status, a fully sequenced genome, transient gut colonization time, and is an efficient cellular factory for heterologous protein production. However, its survivability through the GI tract is low, thus better delivery approaches are being explored to improve its bioavailability. In this study, we employ the incorporation of a double coated mucoadhesive film consisting of sodium alginate and Lycoat RS 720 film as the inner coat. The formulated film exhibits good mechanical properties of tensile strength and percent elongation for manipulation and handling with an entrapment yield of 93.14±2.74%. The formulated mucoadhesive film is subsequently loaded into gelatin capsules with an outer enteric Eudragit L100-55 coating capable of a pH-dependent breakdown above pH 5.5 to protect against gastric digestion. The final product and unprotected controls were subjected to *in vitro* simulated gastrointestinal digestions to assess its survivability. The product demonstrated enhanced protection with an increase of 69.22±0.67% (gastric) and 40.61±8.23% (intestinal) survivability compared to unprotected controls after 6 hours of sequential digestion. This translates to a 3.5 fold increase in overall survivability. Owing to this, the proposed oral delivery system has shown promising potential as a live gastrointestinal vaccine delivery host. Further studies involving *in vivo* gastrointestinal survivability and mice immunization tests are currently being carried out to assess the efficacy of this novel oral delivery system in comparison to parenteral routes.

## Introduction

Oral route administration is one of the most preferred route due to ease of administration, cost effective and high level of patient compliance [1]. It is a form of enteral administration where by drugs or active substances are taken by mouth passing through the intestines and absorbed into the blood stream for systemic circulation. This form of administration relies heavily on mucoadhesion strategies which have recently been actively investigated for drug delivery via oral, buccal, nasal, rectal and vaginal routes [2].

Sodium alginate, a natural polymer from brown seaweed extract is one of the most common natural polymers used in oral films with bioadhesive and film forming properties [3]. Such edible films are strong and hydrophilic in nature, whilst exhibiting water resistance. Previously, it was reported that the mechanical properties of sodium alginate-based bioadhesive films can be improved with the addition of starch [4]. In this respect, a modified pea starch polymer namely Lycoat RS720 was incorporated as a film forming agent in this study.

*L*. *lactis* is a gram positive lactic acid bacterium that is widely used in the food industry for centuries [5]. Recently, genetically modified *L*. *lactis* has demonstrated the potential to be developed into novel therapeutic and prophylactic therapies against a wide range of diseases including bowel cancer [6]. *L*. *lactis* is classified as a generally recognized as safe (GRAS) organism by the Food and Drug Administration (FDA), a non-spore forming and a non-gut colonizing bacterium. Its ability to relatively survive transit through the stomach, form associations with the intestinal epithelium and innate immunomodulatory properties makes *L*. *lactis* an attractive candidate for development as live vehicles to deliver immunogens targeting the intestinal mucosa [7]. Unfortunately, formulating an oral live lactococcal vaccine is challenging as *L*. *lactis* does not have the potential to withstand the harsh gastrointestinal environment, thus resulting in low survivability upon reaching the lower gastrointestinal tract which subsequently affects elicitation of desired immunogenic responses. Hence, current delivery systems can be potentially improved by introducing an outer pH-dependent enteric coating such as Eudragit L100-55 which is a coating polymer made up of methacrylic acid, and only dissolves above a specific trigger pH. This allows maximum protection and delivery efficiency to targeted sites along the gastrointestinal tract.

In this study, a novel approach describing a double coated mucoadhesive film for oral vaccine delivery is reported. This live oral vaccine produced from a nisin-inducible lactococcal recombinant vector pNZ8048 capable of producing mutated K-ras antigens serve as the active component in this formulation. Physical properties in terms of tensile strength, percentage of elongation, thickness, entrapment yield and uniformity of bacterial distribution data are reported. The effectiveness of this oral film formulation was then subjected to *in vitro* evaluation against simulated gastrointestinal digestion, followed by stability and storage condition assessments.

## Materials and methods

### Bacteria culture

In this study, *L*. *lactis* NZ9000 harboring recombinant pNZ8048 plasmid was cultured in M17 broth supplemented with 0.5% glucose (w/v) and 10μg/ml chloramphenicol. Overnight culture with absorbance reading of 1.0 to 1.2 at 600 nm wavelength was used. Bacteria suspension was prepared by harvesting the pellet upon centrifugation at 3900 rpm for 5 minutes at 4 °C. The culture was then washed twice with phosphate buffered saline (PBS) and a total of 5ml resuspended cell suspension in PBS was used in the film making process.

### Preparation of oral film

Oral films were prepared by dissolving various percentages of sodium alginate and Lycoat RS720 that was obtained from Roquette Pte. Ltd. in 5ml distilled water (Table 1). The film solution was poured into 6 well plates wiped with 4% polyvinyl alcohol (PVA) solution and dried at 30 °C for 36 to 48 hours in a convection oven.

**Table 1:** Formulations for oral film optimizations consisting of various sodium alginate and Lycoat RS720 concentrations.

Formulation code	Sodium alginate (%)	Lycoat RS720 (%)	Ratio (w/w)
F1	1%	-	1:0
F2	2%	-	1:0
F3	3%	-	1:0
F4	4%	-	1:0
F5	5%	-	1:0
F6	6%	-	1:0
F7	3%	0%	1:0
F8	3%	0.3%	1:0.1
F9	3%	0.9%	1:0.3
F10	3%	1.5%	1:0.5
F11	3%	2.1%	1:0.7
F12	3%	2.7%	1:0.9
F13	3%	3.3%	1: 1.1
F14	4%	0%	1:0
F15	4%	0.4%	1:0.1
F16	4%	1.2%	1:0.3
F17	4%	2.0%	1:0.5
F18	4%	2.8%	1:0.7
F19	4%	3.6%	1:0.9
F20	4%	4.4%	1: 1.1

### Determination of tensile strength and elongation properties of film

The tensile properties of films (35 mm x 20 mm) were analyzed using tensile tester (Llyod LF Plus,UK) with parameter set at 100 N force load. The films were then stretched at a test speed of 20 mm/sec until its breaking point.

### Determination of mucoadhesive strength

Since Lycoat RS720 was used to improve the bioadhesion of mucoadhesive films, therefore formulations F7 to F18 were subjected to mucoadhesion strength analysis. Films (45 mm x 20 mm) were analyzed using a tensile tester (Llyod LF Plus, UK) with a force load parameter set at 100 N and test speed of 20 mm/sec until the wet film was pulled out completely from the lower grip.

### Thickness and weight of film

Film thickness (mm) was determined using a vernier caliper and 6 random measurements of the film were taken to obtain mean values. The weight of film (g) was taken using an electronic beam balance (Mettler Toledo, USA).

### Scanning electron microscopy (SEM) analysis

*L*. *lactis* loaded film and blank film samples were mounted on metal stubs with double-sided adhesive tape and coated with platinum (Quorum Technologies Q150R S, UK) under vacuum at 15 mA at room temperature. The morphology of *L*. *lactis* loaded film was examined under a scanning electron microscope (JOEL- JSM-7600F, Japan).

### Entrapment yield evaluation

One *L*. *lactis* loaded film, non-enteric coated capsule with *L*. *lactis* film and enteric coated capsule with *L*. *lactis* film were dissolved in 10 ml of PBS respectively. A total of 1 ml of dissolved film was subjected to serial dilution using PBS. Samples from appropriate dilutions were spread on M17 agar supplemented with 0.5% glucose (w/v) and 10μg/ml chloramphenicol for viable cell count (CFU/ml). The entrapment yield was evaluated using the equation below:

$$\text{Entrapment}\;\text{Yield}\;(\%)=\frac{\text{Number}\;\text{of}\;\text{cells}\;\text{recovered}\;\text{from}\;\text{film}/\text{capsule}\;(\text{CFU}/\text{ml})}{\text{Number}\;\text{of}\;\text{cells}\;\text{loaded}\;(\text{CFU}/\text{ml})}\;\text{x}\;100\%$$

### Enteric coating of *Lactococcus lactis* loaded film

The outer enteric coating solution was prepared according to (Table 2). Films were rolled and packed into gelatin capsules (Nashmir Capsule Sdn. Bhd.) for enteric coating. The gelatin capsules were dipped into enteric coating solution for 15 sec and dried at room temperature for 30 minutes. The coating process was repeated 4 times for optimum coating.

**Table 2:** Formulation of enteric coating suspension.

Ingredients	Weight (g)	Function
Eudragit L100-55 co-polymer	3.125g	Polymer
Triethyl citrate	0.312g	Plasticizer
Talcum powder	1.312g	Anti-tacking
Titanium oxide	0.25g	Opaque
Acetone	17.145g	Diluent
Isopropanol	25.71g	Diluent
Water	2.145g	Diluent
Total	50g	-

### *In vitro* simulated gastrointestinal digestion

To evaluate the survivability of *L*. *lactis* in simulated gastric condition, one piece of each enteric coated capsule with *L*. *lactis* film, non-enteric coated capsule with *L*. *lactis* film, *L*. *lactis* film and 1 ml of free bacteria cells were digested in 10 ml filtered SGF containing 2000 U/ml pepsin (Amresco, USA) in sterile PBS at pH 2. Samples were digested in separate tubes at 37 °C and harvested every hour up to 4 hours for survivability evaluation. Digested films, capsules and free bacterial cells were then centrifuged at 3900 rpm for 5 minutes at 4 °C and washed with PBS. Samples were serially diluted with PBS and plated on M17 agar supplemented with 0.5% glucose (w/v) and 10 μg/ml chloramphenicol followed by incubation at 30 °C for 48 hours for viable cell count (CFU/ml). The entire processes was repeated in filtered SIF containing 3mg/ml pancreatin (Sigma Aldrich, USA) and 0.1% (w/v) bile salt (Sigma Aldrich, USA) at pH 8. Sequential digestion was done by digesting samples in SGF for 2 hours followed by SIF for 4 hours. Films, capsules and free bacterial cells without digestion were used as normalization controls. The survivability (%) was determined using the equation below:

$$S\text{urvivability}\;(\%)=\frac{\text{Number}\;\text{of}\;\text{viable}\;\text{count}\;\text{every}\;\text{hour}\;(\text{CFU}/\text{ml})\;}{\text{Number}\;\text{of}\;\text{viable}\;\text{count}\;\text{of}\;\text{control}\;\text{sample}\;(\text{CFU}/\text{ml})\;}\;\text{x}\;100\%$$

### Storage of enteric coated capsules and its survivability

The survivability of *L*. *lactis* at 23 °C, 4 °C and -20 °C were evaluated. One enteric coated capsule containing *L*. *lactis* film was retrieved every week up to 4 months from various storage conditions. The samples were dissolved in 10 ml PBS and serial dilutions were performed. Samples were spread on M17 agar supplemented with 0.5% glucose (w/v) and 10 μg/ml chloramphenicol followed by incubation at 30 °C for 48 hours for viable cell count (CFU/ml). Survivability (%) was determined using the equation below:

$$S\text{urvivability}\;(\%)=\frac{\text{Number}\;\text{of}\;\text{viable}\;\text{count}\;\text{every}\;\text{week}\;(\text{CFU}/\text{ml})\;}{\text{Number}\;\text{of}\;\text{viable}\;\text{count}\;\text{at}\;\text{week}\;0\;(\text{CFU}/\text{ml})\;}\;\text{x}\;100\%$$

### Statistical analysis

All experiments were performed in triplicates unless stated otherwise, and reported as mean ± standard deviation (SD). Data were subjected to statistical significance analysis utilizing two tailed T-test (Microsoft Excel 2013 for Windows) with a threshold value at *p*≤0.05.

## Results & discussions

### Formulations of double coated mucoadhesive film

**Fig 1:**
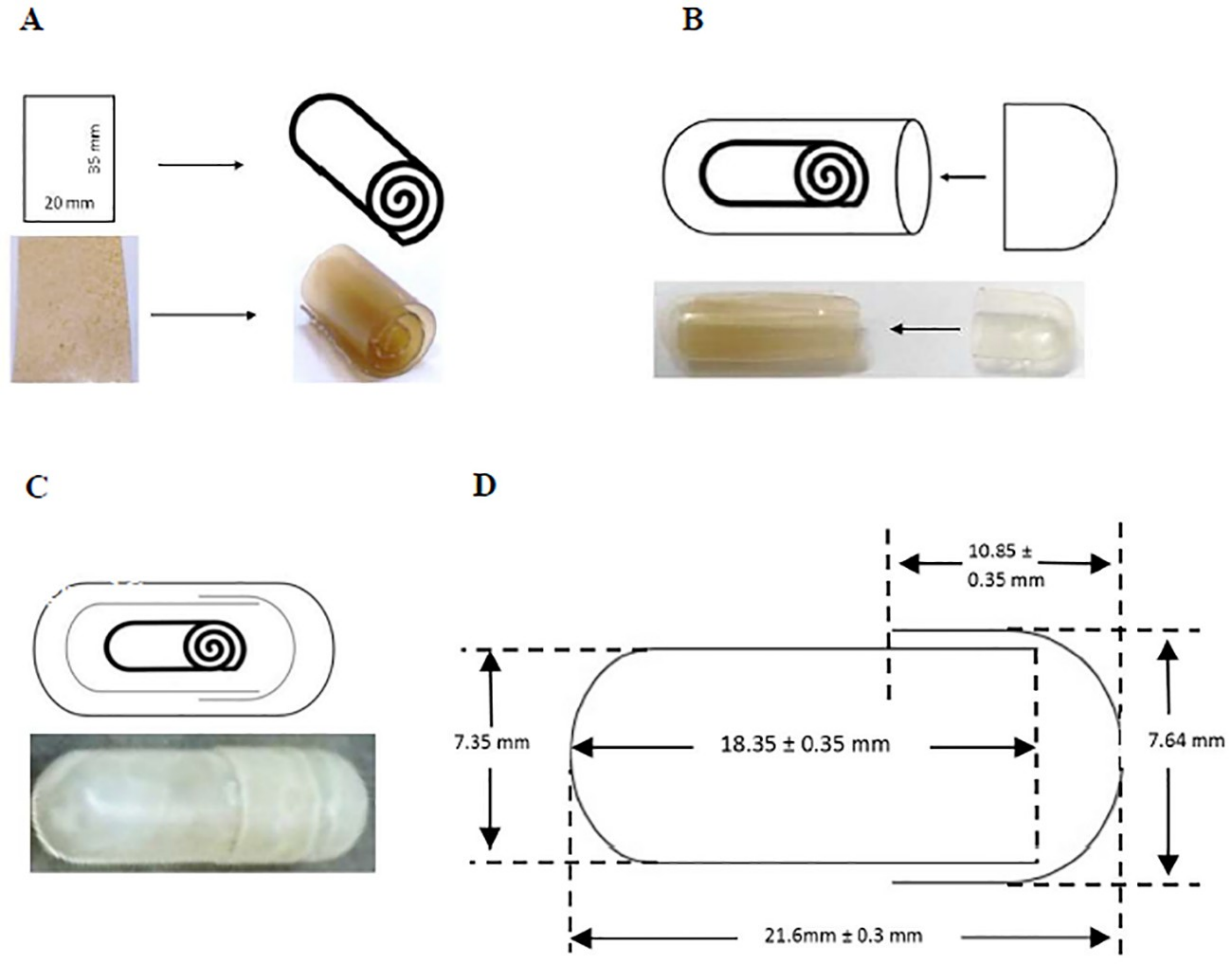
Schematic diagram and product illustrations of the double coated mucoadhesive film. (A-B) Preparation and loading of mucoadhesive film into gelatin capsule. (C) Enteric coating of the gelatin capsule containing the mucoadhesive film. (D) Dimensions of hard gelatin capsule.

Oral films are defined as thin and flexible layers of polymers known for its fast dissolution rate and long residence time at the site of administration [8]. In this study, the appearance of all the products were illustrated at (Fig 1). The *L*. *lactis* mucoadhesive film demonstrated round, flat and smooth surface with brownish in colour (Fig 1A). This formulated mucoadhesive film was loaded with *L*. *lactis* was cut into rectangular shapes and was manually rolled into cylindrical shapes before packing into the body of gelatin capsules with size 0 (Fig 1D). Subsequently, the cap of the capsule was tightly closed and ready for enteric coating process (Fig 1B–1D). Finally, the final product of Eudragit L100-55 coated gelatin capsule containing *Lactococcus lactis* film demonstrated whitish in colour with overall smooth and even coating (Fig 1C).

### Evaluation of physical and mechanical properties of mucoadhesive film formulations

In this study, the formulation of a mucoadhesive oral film containing *L*. *lactis* as a live vector for vaccine delivery was employed. In this respect, selecting the components for mucoadhesive oral film formulation with optimum physical and mechanical properties is essential to ensure effective delivery of biologics. The formulation was first optimized without the addition of Lycoat RS720 based on optimum tensile strength, percentage of elongation, and thickness. Generally, increasing sodium alginate concentrations in film making demonstrated an increase in tensile strength and thickness of the film (Table 3) (S1 Table). However, the percentage of elongation was found to be inversely proportional to sodium alginate concentrations. Ideally, films should not be too flexible and should be ranged between 2.2% to 32.2% as too much elongation during cutting and packaging may cause film variations leading to non-uniform distribution of content [8,9]. While the percentage of elongation was found to be inversely proportional to sodium alginate concentrations, no significant difference was reported against F1 (with the exception of F5), leading to all formulations to fall within the recommended flexibility range (S2 Table).

**Table 3:** Optimization of physical and mechanical properties of mucoadhesive film formulations. All experiments were performed in triplicates unless stated otherwise, and reported as mean ± standard deviation (SD).

Formu-lation	Sodium alginate (%)	Lycoat RS720 (%)	Ratio ^a^ (w/w)	Tensile strength (N)	Mucoadhe-sive strength (N)	Elongation (%)	Wet film elongation (%)	Weight (g)	Thickness (mm)
F1	1%	-	1:0.0	37.28 ± 8.89	NA	6.70 ± 1.20	NA	0.42 ± 0.01	0.010 ± 0.001
F2	2%	-	1:0.0	57.74 ± 16.10	NA	6.50 ± 0.55	NA	0.82 ± 0.03	0.020 ±0.001
F3	3%	-	1:0.0	68.15 ± 12.73	NA	5.50 ± 3.63	NA	1.14 ± 0.01	0.022 ± 0.001
F4	4%	-	1:0.0	62.21 ± 7.07	NA	3.00 ± 1.53	NA	1.50 ± 0.01	0.028 ± 0.001
F5	5%	-	1:0.0	85.74 ± 15.71	NA	3.80 ± 1.54	NA	1.82 ± 0.09	0.034 ± 0.006
F6	6%	-	1:0.0	87.23 ± 4.62	NA	4.80 ± 4.00	NA	2.13 ± 0.05	0.033 ± 0.002
F7	3%	0.0%	1:0.0	NA	0.59 ± 0.17	NA	9.77 ± 1.97	1.31 ± 0.02	0.022 ± 0.002
F8	3%	0.3%	1:0.1	NA	0.11 ± 0.05	NA	12.60 ± 1.39 †	1.27 ± 0.06	0.020 ± 0.002
F9	3%	0.9%	1:0.3	NA	0.35 ± 0.21	NA	12.85 ± 4.24	1.50 ± 0.05	0.026 ± 0.001
F10	3%	1.5%	1:0.5	NA	0.38 ± 0.37	NA	16.94 ± 0.27†	1.71 ± 0.03	0.028 ± 0.002
F11	3%	2.1%	1:0.7	NA	0.52 ± 0.38	NA	17.72 ± 1.79†	1.95 ± 0.09	0.029 ± 0.003
F12	3%	2.7%	1:0.9	NA	0.64 ± 0.83	NA	17.15 ± 5.46	1.99 ± 0.07	0.031 ± 0.004
F13	3%	3.3%	1:1.1	NA	0.88 ± 0.75	NA	14.68 ± 3.24†	2.50 ± 0.10	0.033 ± 0.002
F14	4%	0.0%	1:0.0	NA	0.43 ± 1.41	NA	4.68 ± 1.41	1.52 ± 0.04	0.026 ± 0.002
F15	4%	0.4%	1:0.1	NA	0.36 ± 0.32	NA	12.00 ± 4.15	1.67± 0.10	0.027 ± 0.003
F16	4%	1.2%	1:0.3	NA	0.27 ± 0.20	NA	17.07 ± 2.80†	1.93 ± 0.07	0.032 ± 0.005
F17	4%	2.0%	1:0.5	NA	0.33 ± 0.25	NA	22.19 ± 2.10‡	2.18 ± 0.09	0.032 ± 0.002
F18	4%	2.8%	1:0.7	NA	0.23 ± 0.07	NA	21.96 ± 0.08‡	2.51 ± 0.07	0.036 ± 0.001
F19	4%	3.6%	1:0.9	NA	0.28 ± 0.21	NA	16.59 ± 0.77‡	2.78 ± 0.02	0.040 ± 0.002
F20	4%	4.4%	1:1.1	NA	0.31 ± 0.16	NA	18.32 ± 11.09	3.20 ± 0.08	0.041 ± 0.005

^(†)^ and ^(‡)^ indicates statistically significant difference against F7 and F14 with p≤0.05 and p≤0.01 respectively.

^a^ The formulation ratio (w/w) between sodium alginate and Lycoat RS720

Besides tensile strength and percentage of elongation, measurement of film thickness was also essential to ensure uniformity as it is directly related to dose consistency and provision of sufficient bioadhesion. For instance, a previous study suggested that an ideal film thickness should range between 50 to 1000 μm [10]. However, another study suggested that films with an optimum thickness of 5 to 200 μm could also provide an accurate dose and good absorption [11]. In this study, low concentrations of sodium alginate in F1 and F2 resulted in thin film formation with less than 20 μm thickness, which consequently resulted in handling difficulties during film packing into gelatin capsules for enteric coating (Table 3). On the other extreme, higher concentrations of sodium alginate resulted in thicker film formation with approximately 34 μm, resulting in handling difficulties due to poor flexibility, rigidity and cracking during cutting procedures. As such, 3% and 4% sodium alginate were selected as the optimum thickness for further optimization with 22 μm and 28 μm respectively.

Further mucoadhesion optimization was done with the addition of Lycoat RS720 polymer to the optimized 3% and 4% sodium alginate formulations respectively. The optimization started with a ratio of 1:0.1 up to 1:1.1 with an increase of 1:0.2 per interval. The addition of Lycoat RS720 polymer to both 3% and 4% sodium alginate does not result in an increase of mucoadhesion strength (*p* ≥ 0.05) (S3 Table). Previous studies have reported an optimum mucoadhesive strength of 0.39±0.01 N which is close to the mucoadhesive strength of the F15 formulation (Table 3) [12]. Interestingly, addition of Lycoat RS720 also demonstrated a significant increase in percentage of elongation in both 3% and 4% sodium alginate formulations where *p-value* less than 0.05 (S3 Table). When 3% and 4% formulations were compared, both formulations showed no significant difference towards the percentage of elongation (S3 Table). This means that the addition of Lycoat RS720 did not result in significant mucoadhesive strength changes (*p* ≥ 0.05), but rather, an increases in percentage elongation (*p* ≤ 0.05) (S3 Table). With both physical and mechanical properties identified, the optimum film formulation which is flexible in terms of tensile strength, elongation percentage and optimum thickness for better handling of film was found to be F17 consisting of 4% sodium alginate and 2% Lycoat RS720 (Table 3).

### Surface morphology evaluation of mucoadhesive film using SEM

**Fig 2:**
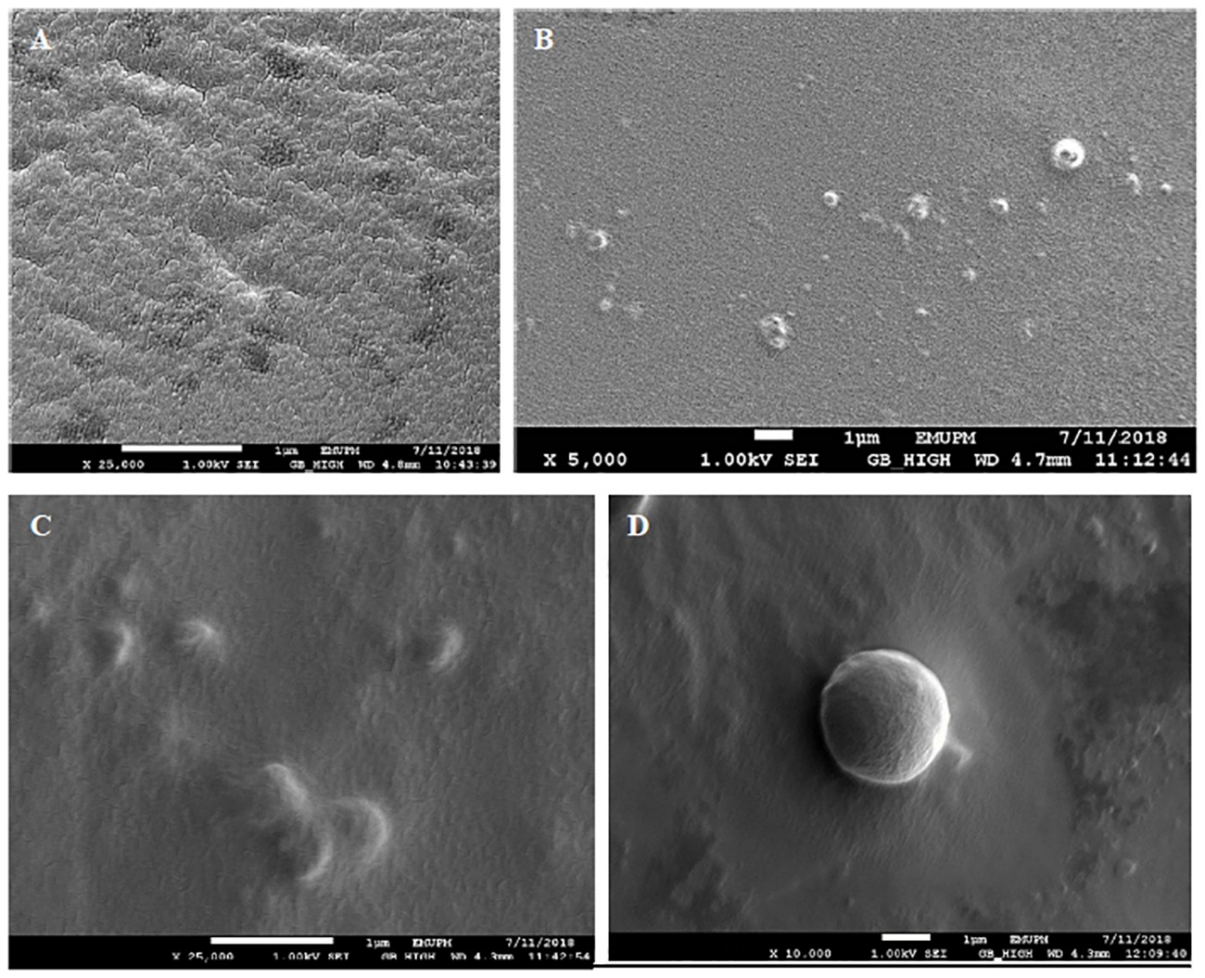
SEM images of mucoadhesive films. Illustrated images show the control film without *L*. *lactis* at 25,000x magnification (A) and mucoadhesive film loaded with *L*. *lactis* (B, C and D) at 5,000x, 10,000x, and 25,000x magnification respectively.

Surface morphology and structural conformation of the film as well as entrapment efficiency were evaluated by SEM. Variations in surface morphology was noticeable between mucoadhesive films with entrapped *L*. *lactis* in comparison to control blank films without *L*. *lactis* (Fig 2A). The control blank film demonstrated a smooth surface overall with several dark spots that may have resulted from uneven coating of platinum during sample preparation procedures. Minor cracks presumably due uneven blending of biopolymers during the process of film formation were also detected for blank films under 25,000 magnification (Fig 2A) [13].

As photographed in (Fig 2B to 2D), mucoadhesive film with *L*. *lactis* demonstrated an uneven and bumpy surface due to embedded bacteria within the biopolymer matrices. A previous study also reported that the morphology of sodium alginate film loaded with *Lactobaccilus plantarum* demonstrated slightly uneven surfaces due to embedded microorganisms within the biopolymer matrices [14]. The bumpy size matches with the size of *L*. *lactis* which is reported to be up to 1.52μm with a spherical or ovoid like morphology [15]. Variation in bump sizes of *L*. *lactis* may be attributed to stress responses due to dehydration of bacterial cells during the film forming processes which was also reported in previous studies [14]. The interaction between the biopolymer matrices and *L*. *lactis* which demonstrates a smooth surface around a single spherical shaped bacterial cell of approximately 1.5μm (Fig 2D) indicated that *L*. *lactis* was successfully distributed and embedded within the biopolymer matrix.

### Entrapment yield of mucoadhesive films containing *L*. *lactis*

The efficiency of *L*. *lactis* being entrapped into mucoadhesive film was evaluated by determining the viability of *L*. *lactis* before and after casting into films using an air convection drying method over 48 hours. *L*. *lactis* was found to retain viability with a relatively high entrapment yield of 93.14 ± 2.74% (Table 4). Interestingly, this approach demonstrated a higher entrapment and *L*. *lactis* survival yield compared to conventional microencapsulation and spray drying methods. Previous study reported that the survival rate of a rifampicin resistant variant of *Lactobacillus paracasei* NFBC 338 and *Lactobacillus rhamnosus GG* was approximately 80% and 60%, respectively during spray-drying in reconstituted skim milk [16,17]. It was suspected that the high entrapment of 93.14 ± 2.74% that was reported in this study may have been attributed to the use of a lower drying temperature at 30°C and a longer adaptation time of 1 day [18].

**Table 4:** The viability of *L*. *lactis* throughout the film forming and enteric coating process.

Type of preparations	Average log CFU/ml ± SD	Yield (%)
Free *L*. *lactis* cells	11.91 ± 0.16	100.00 ± 0.00
*L*. *lactis* film	11.09 ± 0.33	93.14 ± 2.74
*L*. *lactis* film in gelatin capsule	11.08 ± 0.02	93.03 ± 0.20
Eudragit coated capsule containing *L*. *lactis* film	11.01 ± 0.03	92.51 ± 0.29

Additionally, the entrapment yield could theoretically be increased by using *L*. *lactis* harvested from the stationary phase rather than the log phase since cells at stationary phase generally exhibit a higher stress tolerance as a result of triggered stress responses upon the deprivation of nutrients and accumulation of toxic metabolites [19]. Loading of *L*. *lactis* film into gelatin capsules followed by Eudragit L-100-55 enteric coating also showed no significant reduction in viability (Table 4). Therefore, this study provides an alternative approach to conventional microencapsulation techniques with promising survival and entrapment yield of *L*. *lactis*.

### *In vitro* simulated gastrointestinal survival

**Fig 3:**
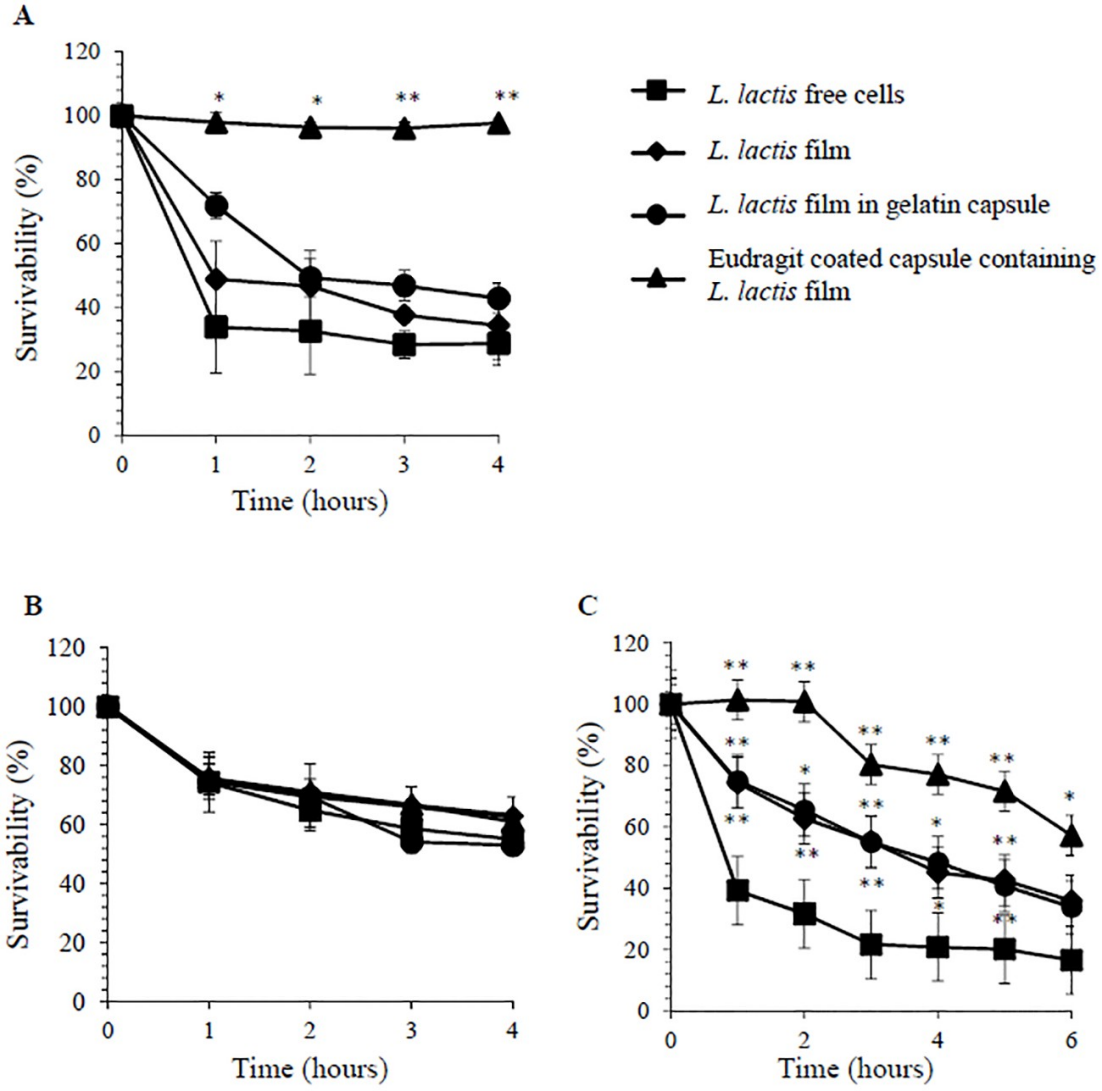
Survivability of *L*. *lactis* in 4 and 6 hours in (A) SGD, (B) SID and (C) SGGD respectively. (*) and (**) indicate statistically significant difference against *L*. *lactis* free cells with *p*≤0.05 and *p*≤0.01 respectively.

Various *L*. *lactis* formulations were assessed individually *in vitro* in SGD and SID to determine the survivability of *L*. *lactis*. The viability of *L*. *lactis* free cell suspension decreased sharply by 8.85 log CFU/ml upon the first hour of digestion in SGF (Table 5). This is best explained by the acid stress responses due to low pH medium used in this study. As lactic acid accumulates, the pH of medium drops, resulting in decreased glycolytic flux and growth rate, eventually compromising cell viability [20]. Acidic pH 2 is considered lethal to *L*. *lactis* which explains the drastic viability drop in the first hour in SGD. However, the subsequent 3 hours of exposure to SGF only resulted in 5.09±9.42% decrease in viability or a mere average reduction of 1.15 log CFU/ml (Fig 3A). Similarly, *L*. *lactis* film and *L*. *lactis* film in gelatin capsule also exhibited a similar trend whereby the survivability decreased sharply upon first hour of SGD (Fig 3A). All samples exhibited a plateau state of viability following the first hour of SGD (Fig 3A), presumably due to activation of acid stress responses resulting in a physiological state of increased tolerance to acidic conditions [21]. Eudragit L100-55 coated capsules containing *L*. *lactis* film demonstrated a promising level of survivability with a 97.93 ± 2.95% viability throughout the 4 hours of SGD (Fig 3A). The Eudragit L100-55 coated capsules were also observed to be physically intact throughout the entire 4 hours of SGD, suggesting that the methacrylic acid—ethyl acrylate pH dependent copolymer was successful in providing gastric protection.

**Table 5:** Normalized survivability of various *L*. *lactis* formulations in SGD, SID and SSGD.

Time (hours)	Average survivability (%) ± SD											
	*L*. *lactis* free cells			*L*. *lactis* film			*L*. *lactis* film in gelatin capsule			Eudragit coated capsule containing *L*. *lactis* film		
	SGD	SID	SSGD	SGD	SID	SSGD	SGD	SID	SSGD	SGD	SID	SSGD
0	100.00 ± 0.00	100.00 ± 0.00	100.00 ± 0.00	100.00 ± 0.00	100.00 ± 0.00	100.00 ± 0.00	100.00 ± 0.00	100.00± 0.00	100.00 ± 0.00	100.00 ± 0.00	100.00 ± 0.00	100.00 ± 0.00
1	33.98 ± 14.40	74.34 ± 10.08	39.24 ± 6.70	48.90 ± 3.39	75.25 ± 5.26	74.40 ± 2.86**	71.92 ± 4.01*	74.59 ±8.15	74.94 ± 3.66**	97.93 ± 2.95*	75.73 ± 7.17	101.38 ± 1.77**
2	32.72 ± 13.54	64.86 ± 7.01	31.61 ± 21.76	46.82 ± 2.09	69.85 ± 10.73	62.73 ± 2.34**	49.36 ± 5.95	69.38 ±1.68	65.50 ± 6.29*	96.37 ± 1.56*	71.03 ± 4.37	100.83 ± 0.67**
3	28.55 ± 4.26	58.62 ± 8.19	21.64 ± 15.07	37.68 ± 3.74	66.35 ± 6.39	55.22 ± 1.91**	46.94 ± 4.79	54.07 ±5.73	54.97 ± 6.28**	96.06 ± 1.88**	66.73 ± 1.57	80.31 ± 6.81**
4	28.89 ± 4.98	55.04 ± 4.07	20.79 ± 15.88	34.50 ± 5.95	62.88 ± 6.39	45.10 ± 1.29*	43.02 ± 4.73*	52.98±10.51	48.41 ± 1.48*	97.67 ± 1.50**	61.00 ± 3.76	77.07 ± 4.53**
5			20.13 ± 13.87			42.54 ± 0.27**			40.77 ± 2.29**			71.56 ± 3.63**
6			16.55 ± 19.12			35.83 ± 6.28			33.78 ± 0.46			57.16 ± 8.23*

(*) and (**) indicates statistically significant difference against L. lactis free cells with p≤0.05 and p≤0.01 respectively.

As for SID, all *L*. *lactis* in different formulations demonstrated about 25% loss in survivability or 4 log CFU/ml reduction upon first hour of SID (Fig 3B). Interestingly, a similar plateau-like profile which occurred in SGD was also observed in SID whereby *L*. *lactis* survivability was not significantly different with *p*>0.05 for the remaining hours of digestion, resulting in an average of 58.06±5.11% survivability at the end of 4 hours SID for all samples (Fig 3B) (S5 Table). In this respect, neither the sodium alginate blend films, gelatin capsules nor the enteric coatings provided sufficient protection against SID. The decrease in *L*. *lactis* survivability was expected following the dissolution of Eudragit L100-55 coating upon exposure to pH 8 of the SIF. Since the principal components of microbial cell membranes are fatty acids and lipids, modifications of microbial cells by the presence of bile in the SIF affects membrane permeability and viability of cells [20]. As a 0.1% bile salt concentration was used in this 4 hours SID study, a survivability of about 55% seems to be more robust compared to what was previously reported in literature with 0.02%-0.08% bile salt concentration [21].

Upon individual digestion in SGF and SIF, the various *L*. *lactis* formulations were assessed in *in vitro* SSGD comprising of 2 hours SGD and subsequently 4 hours SID. Sequential digestion in this study exhibited a similar trend as individual SGD and SID whereby the survivability of free *L*. *lactis* demonstrated a sharp decrease upon first hour in SGD and SID followed by plateau state in subsequent hours (Fig 3C). Despite an expected decrease of *L*. *lactis* survivability, it is observed that survivability of *L*. *lactis* in film and gelatin capsules were significantly higher than *L*. *lactis* free cells throughout the 6 hours of digestions with *p*≤ 0.05 (S5 Table). Eudragit L100-55 coated capsules containing *L*. *lactis* films demonstrated an even higher *L*. *lactis* survivability of 57.16 ± 8.23% (Fig 3C) throughout the whole 6 hours of SSGD. Therefore, this study proved that Eudragit L100-55 coated capsules containing sodium alginate-Lycoat RS720 mucoadhesive films were capable of increasing the gastrointestinal survival of *L*. *lactis* effectively by 3.5 folds compared to *L*. *lactis* free cell controls for lower intestinal targeting of vaccines or other biologics.

### Viability of enteric coated *Lactococcus lactis* under various storage conditions

**Fig 4:**
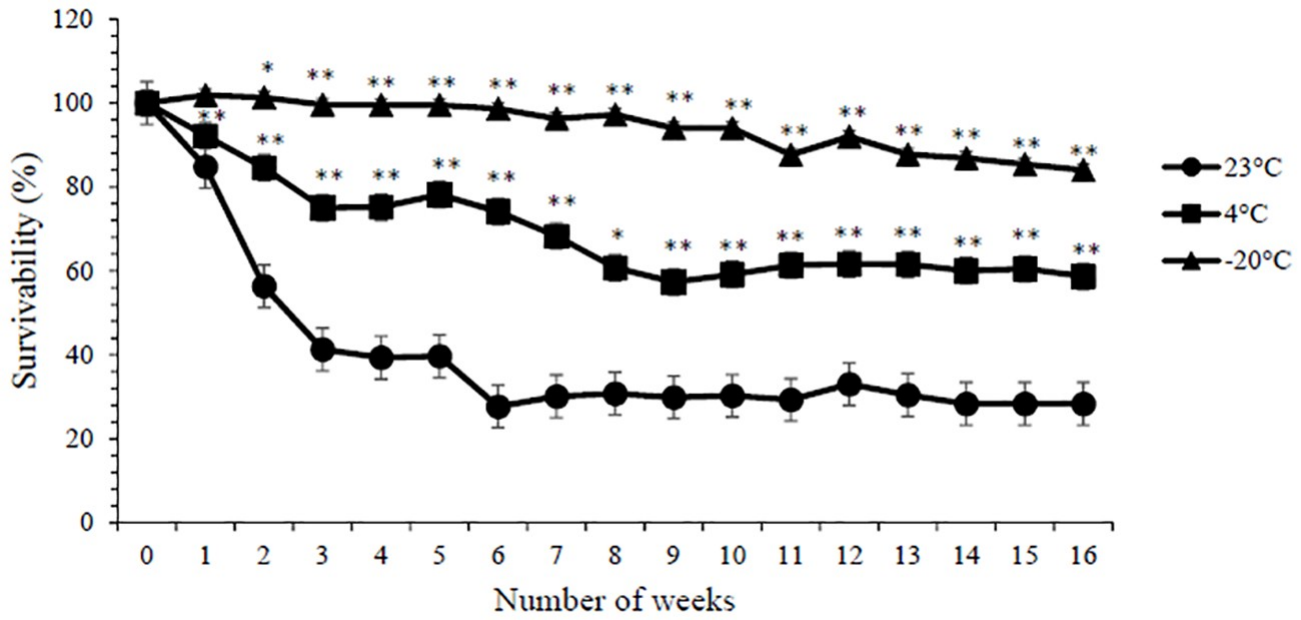
Survivability of *L*. *lactis* at 23°C, 4°C and -20°C storage up to 4 months. (*) and (**) indicate statistically significant difference against *L*. *lactis* stored at 23°C with *p*≤0.05 and *p*≤0.01 respectively.

The stability of enteric coated capsules loaded with *L*. *lactis* film was evaluated weekly up to 4 months following storage at 23 °C, 4 °C, and -20 °C. Generally, *L*. *lactis* was unstable when stored at higher temperatures which explained the gradual decrease in its survivability by 2.5 folds following 3 weeks storage at 23 °C and remained in a plateau state for up to 4 months (Fig 4) (S4 Table). This drastic reduction in cell viability was comparable with *L*. *casei* and *B*. *animalis* when incorporated into whey protein isolate films at 23 °C [22]. *L*. *lactis* stored at 4°C also exhibited a similar trend but with a decrease of only 1.3 folds up to week 3 (Fig 4). It was previously reported that the incorporation of probiotic cell suspension in pullulan film demonstrated an 80% viability in refrigerated condition which is comparable with sodium alginate and Lycoat RS720 blend film used in this study, resulting in 75.07±7.84% *L*. *lactis* viability after 3 weeks [23]. Another study also reported that the drop in cell viability was less accentuated at 4 °C and the cell viability was reduced by only 1 to 2 logs for *L*. *casei* and *B*. *animalis* film [22]. Past literature suggests that the expected shelf life of edible films incorporated with probiotic cultures is 63 to 100 days and 17 to 30 days when stored at 4 °C and 23 °C, respectively. This is reflected in this study where the viability of *L*. *lactis* stored at 23 °C and 4 °C decreased to less than 60% upon the first and third month of storage, respectively. *L*. *lactis* was reported to have the highest stability at -20 °C storage with up to 82% survivability after 4 months of storage. These results suggested that the current probiotic film was also able to sustain a prolonged shelf life at low temperatures, presumably due to a decrease in bacterial metabolism [24].

## Conclusion

This study demonstrates the possibility of developing an orally administered live vaccine delivery approach employing recombinant *L*. *lactis* with enhanced survivability in the form of a double coated mucoadhesive film. This approach may serve as an alternative to conventional microencapsulation as the entrapment of *L*. *lactis* using air convection drying into an edible mucoadhesive film was able to enhance its viability up to 3.5 folds after 6 hours of digestion compared to unprotected controls. Therefore, this novel double coated mucoadhesive film method may be employed as a potential oral vaccine delivery approach targeting lower intestinal tract immunization with a relatively lower cost and higher compliance compared to existing conventional invasive approaches of administration.

## Supporting information

S1 Table Replicates of tensile strength / mucoadhesive strength (N), percentage of elongation (%), weight (g) and thickness (mm) of films. (DOCX)

S2 Table T-test results of various mucoadhesive film formulations for tensile strengths, elongation, weight, and thickness. (*) and (**) indicates statistically significant difference with *p*≤0.05 and *p*≤0.01 respectively.
(DOCX)

S3 Table T-test results of various mucoadhesive film formulations made up to 3% and 4% (w/v) sodium alginate for mucoadhesive strength, wet film elongation, weight and thickness. (*) and (**) indicates statistically significant difference with *p*≤0.05 and *p*≤0.01 respectively.
(DOCX)

S4 Table Replicates of log CFU/mL and survivability of *L*. *lactis* free cells, *L*. *lactis* films, *L*. *lactis* films in capsules and Eudragit coated capsules containing *L*. *lactis* film in SGD, SID and SSGD respectively. (DOCX)

S5 Table T-test of *L*. *lactis* film, *L*. *lactis* in gelatin capsule, and Eudragit coated capsule containing *L*. *lactis* film against *L*. *lactis* free cells in SGD, SID and SSGD respectively. (*) and (**) indicates statistically significant difference with *p*≤0.05 and *p*≤0.01 respectively.
(DOCX)

## Abbreviations

*B*. *animalis*: *Bifidobacterium animalis*
CFU: colony forming unit
FDA: Food and Drug Administration
GRAS: Generally Recognized As Safe
*L*. *casei*: *Lactobacillus casei*
*L*. *lactis*: *Lactococcus lactis*
MHC: major histocompatibility complex
NICE: nisin controlled expression
PBS: Phosphate buffer saline
PVA: polyvinyl alcohol
RT: room temperature
SD: standard deviations
SEM: scanning electron microscope
SGD: simulated gastric digestion
SGF: simulated gastric fluid
SID: simulated intestinal digestion
SIF: simulated intestinal fluid
SSGD: simulated sequential gastrointestinal digestion
