# Supplementary material for: Enhanced gastrointestinal survivability of recombinant Lactococcus lactis using a double coated mucoadhesive film approach
Source: PLoS One. 2019 Jul 23;14(7):e0219912. doi: 10.1371/journal.pone.0219912 (PMC6650035; doi:10.1371/journal.pone.0219912)
Supplement: S1 Table — (DOCX) [file pone.0219912.s001.docx]

S1 Table: Replicates of tensile strength / mucoadhesive strength (N), percentage of elongation (%), weight (g) and thickness (mm) of films.

|  | | | | | | | | | | | | | | | | |
| --- | --- | --- | --- | --- | --- | --- | --- | --- | --- | --- | --- | --- | --- | --- | --- | --- |
|  | Tensile/mucoadhesive strength (N) | | | | Elongation (%) | | | | Weight (g) | | | | Thickness (mm) | | | |
| Formu-lation | A | B | C | Mean | A | B | C | Mean | A | B | C | Mean | A | B | C | Mean |
| F1 | 37.28 | 30.99 | 43.57 | 37.28±  8.89 | 6.70 | 5.80 | 7.50 | 6.67  ± 1.20 | 0.41 | 0.42 | 0.42 | 0.42 ± 0.01 | 0.013 | 0.012 | 0.011 | 0.010  ±  0.001 |
| F2 | 49.28 | 47.63 | 76.31 | 57.74±  16.10 | 7.10 | 6.17 | 6.13 | 6.47  ± 0.55 | 0.80 | 0.84 | 0.78 | 0.82 ± 0.03 | 0.020 | 0.020 | 0.017 | 0.020  ±  0.001 |
| F3 | 72.74 | 53.76 | 77.95 | 68.15±  12.73 | 9.60 | 2.67 | 4.27 | 5.51  ± 3.63 | 1.14 | 1.14 | 1.13 | 1.14 ± 0.01 | 0.021 | 0.022 | 0.022 | 0.022  ±  0.001 |
| F4 | 57.22 | 59.11 | 70.30 | 62.21±  7.07 | 4.70 | 1.70 | 2.67 | 3.02  ± 1.53 | 1.50 | 1.50 | 1.52 | 1.51 ± 0.01 | 0.028 | 0.028 | 0.025 | 0.028  ±  0.001 |
| F5 | 67.65 | 93.69 | 95.90 | 85.74±  15.71 | 3.57 | 2.33 | 5.40 | 3.77  ± 1.54 | 1.88 | 1.75 | 1.67 | 1.82 ± 0.09 | 0.038 | 0.030 | 0.037 | 0.034  ±  0.006 |
| F6 | 82.05 | 88.73 | 90.92 | 87.23±  4.62 | 2.47 | 9.47 | 2.60 | 4.85  ± 4.00 | 2.16 | 2.09 | 2.17 | 2.13 ± 0.05 | 0.033 | 0.033 | 0.035 | 0.033  ±  0.001 |
| F7 | 0.49 | 0.50 | 0.78 | 0.59  ±  0.17 | 7.63 | 10.17 | 11.50 | 9.77  ± 1.97 | 1.29 | 1.33 | 1.30 | 1.31± 0.02 | 0.023 | 0.023 | 0.020 | 0.022  ±  0.002 |
| F8 | 0.15 | 0.13 | 0.05 | 0.11  ±  0.05 | 11.00 | 13.40 | 13.40 | 12.60±  1.39 | 1.27 | 1.29 | 1.24 | 1.27 ± 0.06 | 0.018 | 0.023 | 0.018 | 0.020  ±  0.002 |
| F9 | 0.11 | 0.41 | 0.52 | 0.35  ±  0.21 | 10.40 | 17.75 | 10.40 | 12.85±  4.24 | 1.53 | 1.53 | 1.44 | 1.5 ± 0.05 | 0.023 | 0.027 | 0.027 | 0.026  ±  0.001 |
| F10 | 0.19 | 0.14 | 0.80 | 0.38 ± 0.37 | 16.63 | 17.10 | 17.10 | 16.94±  0.27 | 1.73 | 1.71 | 1.68 | 1.71 ± 0.03 | 0.023 | 0.030 | 0.032 | 0.028  ± 0.002 |
| F11 | 0.12 | 0.58 | 0.87 | 0.52  ± 0.38 | 18.75 | 15.65 | 18.75 | 17.72±  1.79 | 1.99 | 1.99 | 1.88 | 1.95 ± 0.09 | 0.025 | 0.028 | 0.033 | 0.029  ± 0.003 |
| F12 | 0.06 | 0.28 | 1.59 | 0.64 ± 0.83 | 20.30 | 20.30 | 10.85 | 17.15±  5.46 | 2.16 | 2.19 | 1.62 | 1.99 ± 0.07 | 0.037 | 0.030 | 0.025 | 0.031  ± 0.004 |
| F13 | 0.12 | 0.90 | 1.62 | 0.88  ± 0.75 | 10.93 | 16.55 | 16.55 | 14.68±  3.24 | 2.49 | 2.54 | 2.46 | 2.50 ± 0.10 | 0.030 | 0.032 | 0.038 | 0.033  ±  0.002 |
| F14 | 0.19 | 0.13 | 0.99 | 0.43  ±  0.48 | 3.40 | 4.45 | 6.20 | 4.68  ± 1.41 | 1.53 | 1.48 | 1.56 | 1.52± 0.04 | 0.027 | 0.023 | 0.027 | 0.026  ±  0.002 |
| F15 | 0.15 | 0.73 | 0.21 | 0.36 ± 0.32 | 14.80 | 13.97 | 7.23 | 12.00±  4.15 | 1.70 | 1.75 | 1.56 | 1.67± 0.10 | 0.025 | 0.030 | 0.027 | 0.027  ± 0.003 |
| F16 | 0.13 | 0.50 | 0.19 | 0.27 ± 0.20 | 17.75 | 14.00 | 19.47 | 17.07±  2.80 | 2.00 | 1.94 | 1.86 | 1.93 ± 0.07 | 0.030 | 0.038 | 0.028 | 0.032  ±  0.005 |
| F17 | 0.17 | 0.21 | 0.61 | 0.33 ± 0.25 | 23.40 | 19.77 | 23.40 | 22.19±  2.10 | 2.25 | 2.22 | 2.08 | 2.18 ± 0.09 | 0.032 | 0.033 | 0.030 | 0.032  ±  0.002 |
| F18 | 0.15 | 0.30 | 0.23 | 0.23 ± 0.07 | 21.87 | 22.00 | 22.00 | 21.96±  0.08 | 2.54 | 2.57 | 2.43 | 2.51 ± 0.07 | 0.035 | 0.037 | 0.035 | 0.036  ±  0.001 |
| F19 | 0.09 | 0.51 | 0.24 | 0.28 ± 0.21 | 15.70 | 17.03 | 17.03 | 16.59±  0.77 | 2.77 | 2.80 | 2.77 | 2.78 ± 0.02 | 0.040 | 0.042 | 0.038 | 0.04  ± 0.002 |
| F20 | 0.12 | 0.38 | 0.41 | 0.31 ± 0.16 | 23.35 | 26.00 | 5.60 | 18.32±  11.09 | 3.26 | 3.22 | 3.11 | 3.20 ± 0.08 | 0.040 | 0.047 | 0.037 | 0.041  ± 0.005 |
