# Supplementary material for: Enhanced gastrointestinal survivability of recombinant Lactococcus lactis using a double coated mucoadhesive film approach
Source: PLoS One. 2019 Jul 23;14(7):e0219912. doi: 10.1371/journal.pone.0219912 (PMC6650035; doi:10.1371/journal.pone.0219912)
Supplement: S2 Table — (*) and (**) indicates statistically significant difference with p≤0.05 and p≤0.01 respectively. (DOCX) [file pone.0219912.s002.docx]

| Tensile strength (N) | *p*-value | | | | | | |
| --- | --- | --- | --- | --- | --- | --- | --- |
|  | Formulation code | F1 | F2 | F3 | F4 | F5 | F6 |
|  | F1 |  |  |  |  |  |  |
|  | F2 | 0.083 |  |  |  |  |  |
|  | F3 | 0.017* | 0.258 |  |  |  |  |
|  | F4 | 0.010** | 0.491 | 0.432 |  |  |  |
|  | F5 | 0.037* | 0.090 | 0.308 | 0.308 |  |  |
|  | F6 | 0.006** | 0.064 | 0.140 | 0.011* | 0.839 |  |
| Elongation (%) |  | F1 | F2 | F3 | F4 | F5 | F6 |
|  | F1 |  |  |  |  |  |  |
|  | F2 | 0.764 |  |  |  |  |  |
|  | F3 | 0.626 | 0.647 |  |  |  |  |
|  | F4 | 0.050 | 0.029* | 0.178 |  |  |  |
|  | F5 | 0.020* | 0.112 | 0.509 | 0.574 |  |  |
|  | F6 | 0.576 | 0.580 | 0.884 | 0.609 | 0.759 |  |
| Weight (g) |  | F1 | F2 | F3 | F4 | F5 | F6 |
|  | F1 |  |  |  |  |  |  |
|  | F2 | 0.002** |  |  |  |  |  |
|  | F3 | <0.001** | 0.002** |  |  |  |  |
|  | F5 | 0.002** | 0.004** | 0.009** | 0.060 |  |  |
|  | F6 | <0.001** | 0.001** | 0.001** | 0.001** | 0.030* |  |
| Thickness (mm) |  | F1 | F2 | F3 | F4 | F5 | F6 |
|  | F1 |  |  |  |  |  |  |
|  | F2 | 0.012* |  |  |  |  |  |
|  | F3 | 0.001** | 0.157 |  |  |  |  |
|  | F4 | 0.003** | 0.042* | 0.047* |  |  |  |
|  | F5 | 0.010** | 0.035* | 0.039* | 0.120 |  |  |
|  | F6 | 0.001** | 0.013* | 0.002** | 0.057 | 0.625 |  |

S2 Table: T-test results of various mucoadhesive film formulations for tensile strengths, elongation, weight, and thickness. (*) and (**) indicates statistically significant difference with *p*≤0.05 and *p*≤0.01 respectively.
