# Supplementary material for: Enhanced gastrointestinal survivability of recombinant Lactococcus lactis using a double coated mucoadhesive film approach
Source: PLoS One. 2019 Jul 23;14(7):e0219912. doi: 10.1371/journal.pone.0219912 (PMC6650035; doi:10.1371/journal.pone.0219912)
Supplement: S3 Table — (*) and (**) indicates statistically significant difference with p≤0.05 and p≤0.01 respectively. (DOCX) [file pone.0219912.s003.docx]

| Formulation Code | *p*-value | | | |
| --- | --- | --- | --- | --- |
|  | Mucoadhesive strength (N) | Wet film elongation (%) | Weight (g) | Thickness (mm) |
|  | F7 | F7 | F7 | F7 |
| F7 |  |  |  |  |
| F8 | 0.062 | 0.026* | 0.074 | 0.243 |
| F9 | 0.097 | 0.344 | 0.022* | 0.191 |
| F10 | 0.213 | 0.019* | 0.002** | 0.198 |
| F11 | 0.696 | 0.041* | 0.003** | 0.179 |
| F12 | 0.911 | 0.212 | 0.064 | 0.083 |
| F13 | 0.502 | 0.031* | ≤0.001** | 0.079 |
|  | F14 | F14 | F14 | F14 |
| F14 |  |  |  |  |
| F15 | 0.876 | 0.149 | 0.204 | 0.603 |
| F16 | 0.682 | 0.013* | 0.018* | 0.284 |
| F17 | 0.529 | 0.006** | 0.011* | 0.107 |
| F18 | 0.538 | 0.002** | 0.004** | 0.038* |
| F19 | 0.678 | 0.002** | 0.001** | 0.027* |
| F20 | 0.645 | 0.196 | 0.001** | 0.067 |
|  |  | | | |
| F7 vs F14 | 0.476 | 0.008** | 0.024* | 0.212 |
| F8 vs F15 | 0.289 | 0.857 | 0.011* | 0.009** |
| F9 vs F16 | 0.633 | 0.403 | 0.002** | 0.164 |
| F10 vs F17 | 0.600 | 0.056 | 0.006** | 0.406 |
| F11 vs F18 | 0.265 | 0.057 | ≤0.001** | 0.109 |
| F12 vs F19 | 0.541 | 0.884 | 0.048* | 0.092 |
| F13 vs F20 | 0.244 | 0.669 | 0.003** | 0.233 |

S3 Table: T-test results of various mucoadhesive film formulations made up to 3% and 4% (w/v) sodium alginate for mucoadhesive strength, wet film elongation, weight and thickness. (*) and (**) indicates statistically significant difference with *p*≤0.05 and *p*≤0.01 respectively.
