# Supplementary material for: Enhanced gastrointestinal survivability of recombinant Lactococcus lactis using a double coated mucoadhesive film approach
Source: PLoS One. 2019 Jul 23;14(7):e0219912. doi: 10.1371/journal.pone.0219912 (PMC6650035; doi:10.1371/journal.pone.0219912)
Supplement: S4 Table — (DOCX) [file pone.0219912.s004.docx]

S4 Table: Replicates of Log CFU/mL and survivability of *L. lactis* free cells, *L. lactis* films, *L. lactis* films in capsules and Eudragit coated capsules containing *L. lactis* film in SGD, SID and SSGD respectively.

| *In vitro* | Formulation of *L. lactis* | Hours | 0 | | 1 | | 2 | | 3 | | 4 | | 5 | | 6 | |
| --- | --- | --- | --- | --- | --- | --- | --- | --- | --- | --- | --- | --- | --- | --- | --- | --- |
|  |  | Repli-cates | Log CFU/ml | Surviva-bility | Log CFU/ml | Surviva-bility | Log CFU/ml | Surviva-bility | Log CFU/ml | Survivabili-ty | Log CFU/ml | Survivabi-lity | Log CFU/ml | Survivabi-lity | Log CFU/ml | Survivabi-lity |
| SGD | *L. lactis* free cells | A | 15.28 | 100.00 | 4.45 | 25.76 | 4.03 | 23.33 | 4.60 | 26.61 | 3.54 | 23.15 |  | | | |
|  |  | B | 14.09 | 100.00 | 3.91 | 25.58 | 4.06 | 26.60 | 3.91 | 25.60 | 4.53 | 32.15 |  |  |  |  |
|  |  | C | 12.66 | 100.00 | 7.13 | 50.61 | 6.80 | 48.25 | 4.23 | 33.44 | 3.97 | 32.36 |  |  |  |  |
|  |  | Mean | 14.01 ± 1.31 | 100.00 ±  0.00 | 5.16 ± 1.73 | 33.98  ± 14.40 | 4.96 ± 1.59 | 32.72  ± 13.54 | 4.25  ± 0.34 | 28.55 ±  4.26 | 4.01 ± 0.50 | 28.89 ± 4.98 |  |  |  |  |
|  | *L. lactis* film | A | 13.64 | 100.00 | 6.79 | 49.76 | 6.48 | 47.49 | 4.90 | 35.93 | 4.30 | 31.56 |  |  |  |  |
|  |  | B | 14.48 | 100.00 | 6.54 | 45.16 | 6.44 | 44.48 | 5.09 | 35.14 | 4.43 | 30.59 |  |  |  |  |
|  |  | C | 12.98 | 100.00 | 6.72 | 51.78 | 6.29 | 48.49 | 5.45 | 41.97 | 5.37 | 41.34 |  |  |  |  |
|  |  | Mean | 13.70 ± 0.75 | 100.00 ±  0.00 | 6.68 ± 0.13 | 48.90  ±  3.39 | 6.40 ± 0.10 | 46.82  ±  2.09 | 5.14  ±  0.28 | 37.68 ±  3.74 | 4.70 ± 0.58 | 34.50 ± 5.95 |  |  |  |  |
|  | *L. lactis* in gelatin capsule | A | 13.75 | 100.00 | 9.25 | 67.30 | 5.86 | 42.60 | 5.81 | 42.27 | 5.31 | 38.58 |  |  |  |  |
|  |  | B | 11.16 | 100.00 | 8.31 | 74.42 | 6.00 | 53.77 | 5.21 | 46.69 | 4.74 | 42.48 |  |  |  |  |
|  |  | C | 11.49 | 100.00 | 8.50 | 74.04 | 5.94 | 51.72 | 5.95 | 51.84 | 5.51 | 47.99 |  |  |  |  |
|  |  | Mean | 12.13 ± 1.41 | 100.00 ±  0.00 | 8.69 ± 0.50 | 71.92  ±  4.01 | 5.93 ± 0.07 | 49.36  ±  5.95 | 5.66  ±  0.39 | 46.94 ±  4.79 | 5.19 ± 0.40 | 43.02 ± 4.73 |  |  |  |  |
|  | Eudragit coated capsule contain-ing *L. lactis* film | A | 11.40 | 100.00 | 11.05 | 96.93 | 10.86 | 95.30 | 10.62 | 93.20 | 10.82 | 94.90 |  |  |  |  |
|  |  | B | 12.57 | 100.00 | 12.31 | 97.92 | 11.90 | 94.70 | 12.18 | 96.90 | 12.26 | 97.50 |  |  |  |  |
|  |  | C | 13.97 | 100.00 | 13.82 | 98.93 | 13.84 | 99.10 | 13.70 | 98.10 | 13.63 | 97.60 |  |  |  |  |
|  |  | Mean | 12.65 ± 1.29 | 100.00 ±  0.00 | 12.39 ± 1.39 | 97.93  ±  2.95 | 12.19 ± 1.51 | 96.37  ±  1.56 | 12.17 ±  1.54 | 96.06 ±  1.88 | 12.24 ± 1.41 | 97.67 ±  1.5 |  |  |  |  |
| SID | *L. lactis* free cells | A | 12.09 | 100.00 | 9.55 | 79.00 | 8.55 | 70.76 | 8.21 | 67.92 | 7.19 | 59.52 |  |  |  |  |
|  |  | B | 14.44 | 100.00 | 11.73 | 81.25 | 9.63 | 66.71 | 8.01 | 55.47 | 7.79 | 53.99 |  |  |  |  |
|  |  | C | 15.69 | 100.00 | 9.85 | 62.77 | 8.96 | 57.10 | 8.23 | 52.46 | 8.10 | 51.59 |  |  |  |  |
|  |  | Mean | 14.07 ± 1.83 | 100.00 ±  0.00 | 10.38 ± 1.18 | 74.34  ±  10.08 | 9.05 ± 0.54 | 64.86  ±  7.02 | 8.15  ±  0.12 | 58.62 ±  8.2 | 7.69 ± 0.46 | 55.03 ± 4.07 |  |  |  |  |
|  | *L. lactis* film | A | 13.27 | 100.00 | 10.66 | 80.38 | 10.88 | 82.04 | 9.78 | 73.71 | 9.31 | 70.15 |  |  |  |  |
|  |  | B | 12.75 | 100.00 | 8.91 | 69.87 | 8.37 | 65.65 | 8.04 | 63.06 | 7.70 | 60.34 |  |  |  |  |
|  |  | C | 12.15 | 100.00 | 9.17 | 75.51 | 7.52 | 61.86 | 7.57 | 62.28 | 7.07 | 58.16 |  |  |  |  |
|  |  | Mean | 12.72 ± 0.56 | 100.00 ±  0.00 | 9.58 ± 0.94 | 75.32  ±  5.26 | 8.92 ± 1.75 | 70.14  ± 10.73 | 8.46  ±  1.16 | 66.51 ±  6.39 | 8.02 ± 1.16 | 63.06 ± 6.39 |  |  |  |  |
|  | *L. lactis* in gelatin capsule | A | 12.09 | 100.00 | 9.26 | 76.54 | 9.02 | 74.56 | 7.26 | 60.00 | 5.95 | 49.19 |  |  |  |  |
|  |  | B | 12.86 | 100.00 | 10.36 | 80.55 | 8.49 | 65.99 | 6.82 | 53.00 | 7.70 | 59.85 |  |  |  |  |
|  |  | C | 13.99 | 100.00 | 9.33 | 66.69 | 9.46 | 67.59 | 6.89 | 49.20 | 6.98 | 49.89 |  |  |  |  |
|  |  | Mean | 12.98 ± 0.96 | 100.00  ±  0.00 | 9.65 ± 0.62 | 74.59  ±  7.14 | 8.99 ± 0.49 | 69.38  ±  4.55 | 6.99  ± 0.24 | 54.07 ±  5.48 | 6.88 ± 0.88 | 52.98 ± 5.96 |  |  |  |  |
|  | Eudragit coated capsule contain-ing *L. lactis* film | A | 11.66 | 100.00 | 9.48 | 81.33 | 7.90 | 67.75 | 7.33 | 62.85 | 7.47 | 64.12 |  |  |  |  |
|  |  | B | 13.21 | 100.00 | 9.20 | 69.62 | 8.80 | 66.65 | 8.67 | 65.63 | 7.48 | 56.65 |  |  |  |  |
|  |  | C | 11.95 | 100.00 | 10.49 | 87.78 | 9.89 | 82.76 | 9.29 | 77.73 | 7.50 | 62.76 |  |  |  |  |
|  |  | Mean | 12.27 ± 0.83 | 100.00 ±  0.00 | 9.72 ± 0.68 | 79.58  ±  9.2 | 8.86 ± 1.00 | 72.39  ±  9.00 | 8.43  ±  1.00 | 68.74 ±  7.9 | 7.49 ± 0.01 | 61.17 ± 3.98 |  |  |  |  |
| SSGD | *L. lactis* free cells | A | 16.86 | 100.00 | 5.48 | 32.50 | 5.98 | 35.47 | 3.51 | 20.82 | 2.30 | 13.64 | 3.23 | 19.16 | 2.74 | 16.25 |
|  |  | B | 14.76 | 100.00 | 7.62 | 51.63 | 3.54 | 23.98 | 4.10 | 27.78 | 4.18 | 28.32 | 4.17 | 28.25 | 3.21 | 21.75 |
|  |  | C | 15.89 | 100.00 | 5.45 | 39.48 | 5.25 | 30.83 | 3.02 | 22.53 | 3.77 | 21.90 | 1.99 | 19.98 | 1.68 | 16.19 |
|  |  | Mean | 15.84 ± 0.68 | 100.00 ±  0.00 | 6.18 ± 0.78 | 39.24  ±  6.70 | 4.92 ± 3.40 | 31.61  ± 21.76 | 3.37  ±  2.35 | 21.64 ± 15.07 | 3.21 ± 2.40 | 20.79 ± 15.88 | 3.12 ± 2.13 | 20.13  ± 13.87 | 2.54 ± 2.94 | 16.55 ± 19.12 |
|  | *L. lactis* film | A | 14.62 | 100.00 | 10.97 | 74.99 | 9.21 | 63.02 | 8.51 | 58.18 | 6.85 | 46.86 | 6.13 | 41.92 | 4.79 | 32.77 |
|  |  | B | 15.13 | 100.00 | 11.53 | 76.18 | 8.97 | 59.25 | 8.09 | 53.45 | 7.19 | 47.49 | 6.55 | 43.28 | 6.18 | 40.85 |
|  |  | C | 14.84 | 100.00 | 10.69 | 72.01 | 9.79 | 65.92 | 8.02 | 54.04 | 6.08 | 40.96 | 6.30 | 42.42 | 5.03 | 33.87 |
|  |  | Mean | 14.87 ± 0.26 | 100.00 ±  0.00 | 11.06 ± 0.43 | 74.40  ±  2.86 | 9.32 ± 0.42 | 62.73  ±  2.34 | 8.21  ±  0.26 | 55.22 ±  1.91 | 6.71 ± 0.57 | 45.10 ± 1.29 | 6.32 ± 0.21 | 42.54  ±  0.27 | 5.33 ± 0.74 | 35.83 ± 6.28 |
|  | *L. lactis* in gelatin capsule | A | 10.00 | 100.00 | 7.08 | 70.83 | 5.86 | 58.62 | 4.85 | 48.55 | 4.92 | 49.20 | 4.21 | 42.12 | 3.42 | 34.24 |
|  |  | B | 14.29 | 100.00 | 11.12 | 77.84 | 9.56 | 66.92 | 7.89 | 55.25 | 6.67 | 46.71 | 6.01 | 42.06 | 4.83 | 33.78 |
|  |  | C | 14.17 | 100.00 | 10.79 | 76.15 | 10.05 | 70.96 | 8.66 | 61.11 | 6.99 | 49.33 | 5.40 | 38.13 | 4.72 | 33.32 |
|  |  | Mean | 12.82 ± 2.44 | 100.00 ±  0.00 | 9.66 ± 2.24 | 74.94  ±  3.66 | 8.49 ± 2.29 | 65.50  ±  6.29 | 7.13  ±  2.01 | 54.97 ±  6.28 | 6.19 ± 1.11 | 48.41 ± 1.48 | 5.21 ± 0.91 | 40.77  ±  2.29 | 4.32 ± 0.78 | 33.78 ± 0.46 |
|  | Eudragit coated capsule contain-ing *L. lactis* film | A | 13.95 | 100.00 | 14.05 | 100.75 | 14.20 | 101.81 | 10.97 | 78.67 | 10.58 | 75.81 | 9.98 | 71.56 | 8.70 | 62.37 |
|  |  | B | 14.98 | 100.00 | 14.68 | 98.01 | 14.69 | 98.06 | 12.30 | 82.10 | 11.40 | 76.09 | 10.85 | 72.41 | 8.30 | 55.40 |
|  |  | C | 14.93 | 100.00 | 15.73 | 105.37 | 15.32 | 102.62 | 11.97 | 80.17 | 11.84 | 79.30 | 10.56 | 70.71 | 8.02 | 53.72 |
|  |  | Mean | 14.62 ± 0.58 | 100.00 ±  0.00 | 14.82 ± 0.85 | 101.38 ±  1.77 | 14.74 ± 0.56 | 100.83 ±  0.67 | 11.75 ± 0.69 | 80.31 ± 6.81 | 11.27 ± 0.64 | 77.07 ± 4.53 | 10.46 ± 0.44 | 71.56 ± 3.63 | 8.34 ± 0.34 | 57.16 ± 8.23 |
