# Supplementary material for: Enhanced gastrointestinal survivability of recombinant Lactococcus lactis using a double coated mucoadhesive film approach
Source: PLoS One. 2019 Jul 23;14(7):e0219912. doi: 10.1371/journal.pone.0219912 (PMC6650035; doi:10.1371/journal.pone.0219912)
Supplement: S5 Table — (*) and (**) indicates statistically significant difference with p≤0.05 and p≤0.01 respectively. (DOCX) [file pone.0219912.s005.docx]

S5 Table: T-test of *L. lactis* film, *L. lactis* in gelatin capsule, and Eudragit coated capsule containing *L. lactis* film against *L. lactis* free cells in SGD, SID and SSGD respectively. (*) and (**) indicates statistically significant difference with *p*≤0.05 and *p*≤0.01 respectively.

|  |  | *p*-value | | | |
| --- | --- | --- | --- | --- | --- |
| *In vitro* digestion | Hours | *L. lactis* free cells | *L. lactis* film | *L. lactis* in gelatin capsule | Eudragit coated capsule containing *L. lactis* film |
| SGD | 0 |  |  |  |  |
|  | 1 |  | 0.180 | 0.040* | 0.002** |
|  | 2 |  | 0.130 | 0.155 | 0.001** |
|  | 3 |  | 0.257 | 0.069 | ≤0.001** |
|  | 4 |  | 0.279 | 0.024* | ≤0.001** |
| SID | 0 |  |  |  |  |
|  | 1 |  | 0.896 | 0.975 | 0.855 |
|  | 2 |  | 0.537 | 0.338 | 0.265 |
|  | 3 |  | 0.267 | 0.475 | 0.168 |
|  | 4 |  | 0.147 | 0.768 | 0.135 |
| SSGD | 0 |  |  |  |  |
|  | 1 |  | <0.001** | <0.010** | <0.001** |
|  | 2 |  | 0.007** | 0.012* | <0.001** |
|  | 3 |  | 0.001** | 0.005** | <0.001** |
|  | 4 |  | 0.035* | 0.020* | 0.001** |
|  | 5 |  | 0.003** | 0.008** | <0.001** |
|  | 6 |  | 0.302 | 0.349 | 0.043* |
